# Diagnostic accuracy of the appendicitis inflammatory response (AIR) score for acute appendicitis during pregnancy: A systematic review and diagnostic meta-analysis

**DOI:** 10.1016/j.sipas.2026.100367

**Published:** 2026-10-07

**Authors:** Yasaman Tavakoli, Behdad Ghanami, Azad Alimohammadi, Arefeh Motaharnia

**Affiliations:** a Student Research Committee, Medical student, Department of Medicine, Mazandaran University of Medical Science, Sari, Mazandaran, Iran; b School of Medicine, Tehran Medical Science, Islamic Azad University, Tehran, Iran; c School of Medicine, Iran University of Medical Science, Tehran, Iran

**Keywords:** Acute appendicitis, Pregnancy, Appendicitis inflammatory response (AIR) score

## Abstract

**Purpose:**

This diagnostic test accuracy meta-analysis evaluated the accuracy of the Appendicitis Inflammatory Response (AIR) score for acute appendicitis (AA) in pregnant women.

**Methods:**

Following PRISMA guidance, PubMed, Embase, Scopus, and Web of Science were searched from inception through March 2026 for studies providing data to construct 2 × 2 contingency tables. Pooled sensitivity, specificity, and area under the curve (AUC) were estimated using a bivariate random-effects Reitsma model. Methodological quality was assessed using QUADAS-2. Meta-regression explored AIR cutoff, disease prevalence, and reference-standard category as potential sources of heterogeneity.

**Results:**

Five studies met the eligibility criteria. The pooled sensitivity was 66.7% (95% CI: 44.2–83.5%) and specificity was 75.5% (95% CI: 40.0–93.5%), with a false-positive rate of 24.5% (95% CI: 6.5–60.0%). Overall diagnostic accuracy was moderate (AUC = 0.752; normalized pAUC = 0.669). Substantial heterogeneity was observed (Zhou–Dendukuri I² = 75.6%). AIR cutoff significantly moderated specificity (P < 0.001), but not sensitivity (P = 0.911). In a within-study analysis, increasing the cutoff from ≥4 to ≥9 reduced sensitivity from 99.1% to 36.8% and increased specificity from 42.4% to 97.0%.

**Conclusions:**

The AIR score provides moderate accuracy for AA in pregnancy and should be used as an adjunct to clinical assessment rather than as a stand-alone diagnostic tool. Threshold selection contributes to variability in diagnostic performance. Prospective multicenter studies are needed to establish pregnancy-specific cutoffs.

## Highlights

• The Appendicitis Inflammatory Response (AIR) score demonstrated moderate diagnostic accuracy for detecting acute appendicitis during pregnancy (AUC = 0.752).
• Pooled analysis showed a sensitivity of 69% and specificity of 76%, supporting the AIR score as an adjunct rather than a standalone diagnostic tool.
• Further large prospective multicenter studies are needed to establish pregnancy-specific cut-off values and validate the AIR score in pregnant populations.

## 1 Introduction

The Appendicitis Inflammatory Response (AIR) score is a validated tool designed to sharpen the diagnostic work-up of suspected acute appendicitis (AA). It combines seven clinical and laboratory variables—vomiting, right iliac fossa pain, rebound tenderness or muscular guarding, body temperature, white blood cell count, the percentage of polymorphonuclear leukocytes, and C-reactive protein (CRP)—into a single 12-point score [1,2]. Patients are then sorted into three probability bands: low (0–4 points), intermediate (5–8 points), and high (9–12 points) [1,3]. These bands are meant to steer management: low-probability patients can typically be observed or followed as outpatients, intermediate-probability patients need further work-up, and high-probability patients should proceed promptly to surgical evaluation [1,4,5]. Because it merges clinical signs with inflammatory markers, the World Society of Emergency Surgery (WSES) has endorsed the AIR score as one of its preferred instruments for the initial assessment of suspected AA [5,6].

Clinical scoring systems for diagnosing AA during pregnancy have been evaluated across multiple studies with varying results. The AIR score showed limited diagnostic accuracy in pregnant patients, with sensitivity ranging from 78.8% to 84.8% and specificity of 36.6% to 41.5% when compared to MRI findings [7]. The AIR score has been specifically validated during pregnancy through nested case-control studies [8]. While these clinical scores may help identify pregnant patients requiring advanced imaging like MRI, their relatively low sensitivity suggests further refinement is needed for optimal diagnostic accuracy in this population [7].

Although the AIR score has shown promising diagnostic performance in the general population, its accuracy in pregnant women remains unclear. Therefore, we conducted a systematic review and diagnostic meta-analysis to evaluate the sensitivity, specificity, and overall diagnostic performance of the AIR score for diagnosing AA during pregnancy.

## 2 Methods

### 2.1 Literature search

This review followed the Preferred Reporting Items for Systematic Reviews and Meta-Analyses of Diagnostic Test Accuracy (PRISMA-DTA) framework [9] and was registered prospectively with PROSPERO (registration no. CRD420251067218) before data collection began.

We searched four databases—PubMed, Embase, Scopus, and Web of Science—from their inception through January 7, 2026. Search terms combined relevant Medical Subject Headings (MeSH) with free-text keywords covering acute appendicitis, pregnancy, the AIR score, and diagnostic accuracy (sensitivity, specificity), linked with the Boolean operators AND/OR; the full search strings for each database appear in the Supplementary Material. Titles, abstracts, and full texts were screened independently by two reviewers (YT and AA), with a third reviewer (BG) adjudicating any disagreements.

### 2.2 Inclusion and exclusion criteria

A study qualified for inclusion if it (1) assessed the AIR score's diagnostic accuracy in pregnant women aged 18 years or older, (2) used histopathology as the reference standard for confirming appendicitis, and (3) supplied enough data—either as a 2 × 2 contingency table of true and false positives/negatives or as directly reported sensitivity and specificity—to allow these metrics to be derived (Table 1).

**Table 1:** Appendicitis Inflammatory Response Score [4]**.**

Variable	Criteria	Score
Vomiting	Present	1
Right lower quadrant pain	Present	1
Rebound tenderness or muscular defense	Mild	1
	Moderate	2
	Strong	3
Body temperature	≥ 38.5 °C	1
White blood cell (WBC) count	10.0–14.9 × 10⁹/L	1
	≥ 15.0 × 10⁹/L	2
Proportion of polymorphonuclear leukocytes	70–84%	1
	≥ 85%	2
C-reactive protein (CRP)	10–49 mg/L	1
	≥ 50 mg/L	2
Total		12

Seven variables are assessed and scored accordingly. After the revision proposed in this report a score 0–3 points suggest low probability, a score 4–8 medium probability and a score 9–12 high probability.

We excluded case reports and case series, narrative or systematic reviews and meta-analyses, conference abstracts, animal studies, duplicate publications, studies conducted in non-pregnant populations, and any study that did not report sufficient diagnostic accuracy data.

### 2.3 Data extraction

Using a standardized extraction form, two reviewers (YT and BG) independently pulled data on first author, publication year, country, study design, sample size, patient age, gestational age, pregnancy trimester, CRP, WBC count, the prevalence of histopathologically confirmed appendicitis, the AIR cut-off applied, sensitivity, specificity, PPV, NPV, overall diagnostic accuracy, and the raw true positive (TP), false positive (FP), false negative (FN), and true negative (TN) counts. Any discrepancies were settled by discussion until consensus was reached.

### 2.4 Quality assessment

The methodological quality of the included studies was independently appraised by two reviewers (BG and AM) with the Quality Assessment of Diagnostic Accuracy Studies-2 (QUADAS-2) tool, which covers four domains: patient selection, index test, reference standard, and flow and timing. Each domain was rated for risk of bias, and the first three were also rated for applicability concerns. A third investigator resolved any disagreements between reviewers through discussion until consensus was reached.

### 2.5 Statistical analysis

To quantify the diagnostic performance of the AIR score for AA during pregnancy, we constructed a 2 × 2 contingency table for each study from the reported true-positive (TP), false-positive (FP), false-negative (FN), and true-negative (TN) counts. When a study reported more than one AIR threshold, only one threshold per study was included in the primary pooled analysis to avoid treating correlated observations from the same participants as independent studies. For Moltubak et al. [8], the ≥9 threshold was retained for the primary analysis, while the ≥4 threshold was reserved for a within-study threshold-effect analysis.

We estimated pooled sensitivity, specificity, and false-positive rate using a bivariate random-effects model following the Reitsma approach and generated hierarchical summary receiver operating characteristic (HSROC) curves to summarize overall diagnostic performance using the area under the curve (AUC) and normalized partial AUC (pAUC). Study-level sensitivity and specificity were additionally displayed using forest plots. As complementary analyses, univariate random-effects models were fitted separately for sensitivity and specificity, using logit-transformed proportions and the DerSimonian–Laird estimator.

Heterogeneity across studies was quantified using I², with estimates obtained using the Zhou–Dendukuri and Holling approaches within the bivariate model. Given the reviewer-identified heterogeneity in diagnostic thresholds, patient selection, disease spectrum, and reference standards, exploratory meta-regression analyses were conducted using study-level AIR cut-off and disease prevalence as continuous moderators. A multivariable model including cut-off and prevalence was also fitted. Reference-standard category (pathology-based versus MRI/proxy-based) was examined as a categorical moderator, including a model combining reference-standard category with AIR cut-off. Descriptive subgroup analyses were performed according to lower cut-offs (5–6) versus high cut-offs (≥9) and according to reference-standard category. The effect of the reference standard was further examined by excluding the Kahana [7] study in a sensitivity analysis. Because only five primary studies were available, these moderator and subgroup analyses were considered exploratory and were interpreted cautiously. The two thresholds reported by Moltubak et al. [8] were also compared within the same cohort to directly assess the threshold-related sensitivity–specificity trade-off. We did not formally assess publication bias using Deeks' funnel plot asymmetry test because the number of included studies was too small for a reliable assessment.

Analyses were carried out in R (version 4.5.2; R Foundation for Statistical Computing, Vienna, Austria) using the mada and metafor packages. Statistical significance was defined as a two-sided P value <0.05.

## 3 Results

### 3.1 Study selection and identification

The study selection process is summarized in the PRISMA flow diagram (Fig. 1). The database searches returned 763 records in total: 119 from PubMed, 256 from Embase, 276 from Scopus, and 112 from Web of Science. After 250 duplicates were removed, 513 records underwent title and abstract screening, from which 488 were excluded. The remaining 25 articles were retrieved for full-text assessment; 20 of these were subsequently excluded because the population was irrelevant (n = 15), the data were unusable (n = 1), or the content was unrelated to the AIR score (n = 4). This left five eligible studies, published between 2020 and 2025, for inclusion in the final analysis (Table 2).

**Fig. 1:**
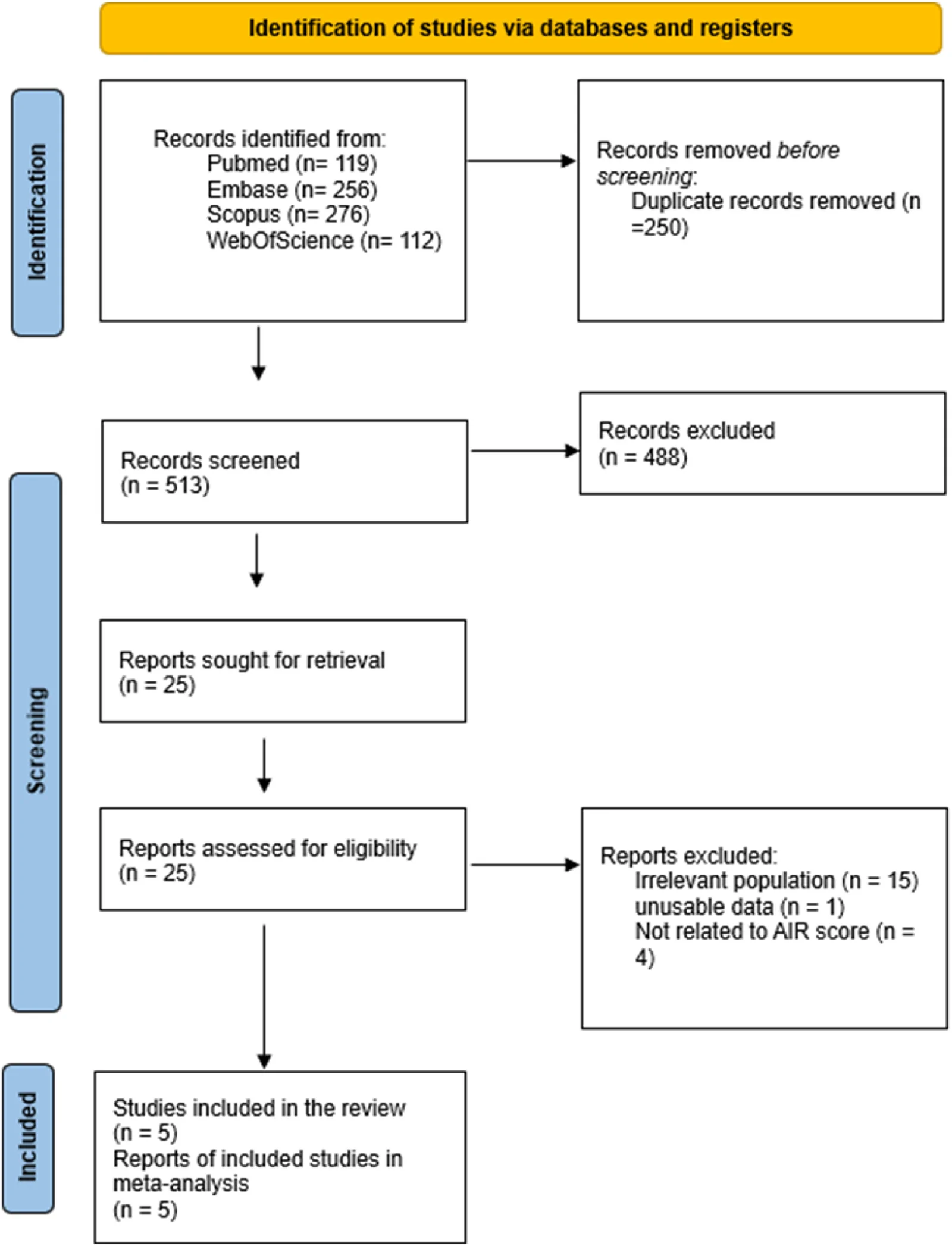
The PRISMA flowchart for the study.

**Table 2:** Characteristics of included studies.

Author (Year)	Country	Design	Sample Number (pregnant)	Comparator	Reference Standard	AIR Cut-off	Sensitivity	Specificity	AUC
Bardakçı et al. (2023) [10]	Turkey	Retrospective observational study	53	Alvarado Score	Pathology results	≥9	85.0%	92.3%	NR
Bekhor et al. (2025) [11]	Israel	Retrospective analysis	180	Alvarado, Tzanakis, RIPASA scores, and MRI	Histopathological evidence	≥5	42.9%	42.4%	NR
Kahana et al. (2024) [7]	Israel	Retrospective cohort study	255	Alvarado Score	MRI findings	≥5	78.8%	41.5%	0.600
Mantoglu et al. (2020) [12]	Turkey	Single-center retrospective study	79	Non-pregnant patients; 8 other scoring systems	Pathology results	≥6	80.0%	71.4%	0.795
Moltubak et al. (2024) [8]	Sweden	Nested case–control study	386	Patients with NSAP	Final diagnosis	≥4	99%	42%	0.88
Moltubak et al. (2024) [8]	Sweden	Nested case–control study	386	Patients with NSAP	Final diagnosis	≥9	37%	97%	0.88

Abbreviations: RIPASA: Raja Isteri Pengiran Anak Saleha Appendicitis Score; AIR: Appendicitis Inflammatory Response Score; MRI: Magnetic Resonance Imaging; AUC: Area Under the Curve; NR: Not Reported.

### 3.2 Characteristics of the included studies

The five included studies, published between 2020 and 2025, were conducted in Turkey (n = 2), Israel (n = 2), and Sweden (n = 1). All studies were observational, including four retrospective studies and one nested case–control study, and sample size ranged from 53 to 386 participants. The included studies differed substantially in how patients were selected: some primarily enrolled pregnant patients who proceeded to surgery for suspected appendicitis, whereas others enrolled patients undergoing an imaging-based diagnostic pathway or included a mixture of operated patients and patients with spontaneous clinical resolution.

Every included study assessed the AIR score in pregnant patients with suspected AA, but the reference standard and diagnostic pathway differed substantially across studies. Bardakçı [10]and Mantoglu [12] used histopathology as the reference standard, restricted to patients who underwent surgery. Moltubak et al. [8] also used histopathology for operated patients, supplemented by a verified non-appendicitis diagnosis for the non-operated comparison group. By contrast, the reference standard was less direct in the remaining two studies: in Kahana et al. [7], the comparator for the non-operated group was the MRI finding itself rather than histopathology, and in Bekhor et al. [11], the non-operated comparator was ascertained by the absence of further healthcare contact rather than by pathology. These differences in reference standard may affect the spectrum and classification of disease and were therefore examined in exploratory analyses. Each study provided extractable TP, FP, FN, and TN data, allowing quantitative synthesis.

AIR cut-offs differed between studies, ranging from ≥5 to ≥9 in the primary pooled dataset. The selected thresholds were ≥9 for Bardakçı, ≥5 for Bekhor, ≥5 for Kahana, ≥6 for Mantoglu, and ≥9 for Moltubak. Reported sensitivity ranged from 36.8% to 85.0%, whereas specificity ranged from 41.4% to 97.0%, demonstrating substantial between-study variation.

Moltubak et al. [8] reported diagnostic performance at two thresholds (≥4 and ≥9) within the same cohort. At the ≥4 threshold, sensitivity was 99.1% and specificity was 42.4%; at ≥9, sensitivity was 36.8% and specificity was 97.0%. Because including both thresholds in the primary meta-analysis would have duplicated participants and violated the assumption of independent studies, only the ≥9 threshold was retained for the primary pooled analysis. The ≥4 threshold was instead used exclusively for the within-study threshold-effect analysis.

During data verification, discrepancies were identified in the extraction of the Bekhor [11] and Mantoglu [12] studies. These data-entry errors were corrected against the corresponding published contingency-table values before the final analyses. All diagnostic measures were recalculated directly from TP, FP, FN, and TN counts to ensure internal consistency.

Thus, the included evidence was characterized by marked differences in diagnostic threshold, patient-selection pathway, disease prevalence, and reference standard, providing a plausible explanation for the substantial heterogeneity observed in the pooled analyses.

### 3.3 Pooled sensitivity and specificity analyses

Forest plots demonstrated considerable variability in the diagnostic performance of the AIR score across the five included studies. Using the bivariate random-effects Reitsma model, the pooled sensitivity was 66.7% (95% CI: 44.2%–83.5%), while the pooled false-positive rate was 24.5% (95% CI: 6.5%–60.0%), corresponding to a pooled specificity of approximately 75.5%. The corresponding univariate random-effects analyses yielded a sensitivity of 66.7% (95% CI: 42.0%–84.7%; I² = 92.0%) and a specificity of 75.5% (95% CI: 46.6%–91.6%; I² = 96.4%), confirming substantial between-study heterogeneity. Fig. 2, Fig. 3 present the forest plots for pooled sensitivity and specificity, respectively.

**Fig. 2:**
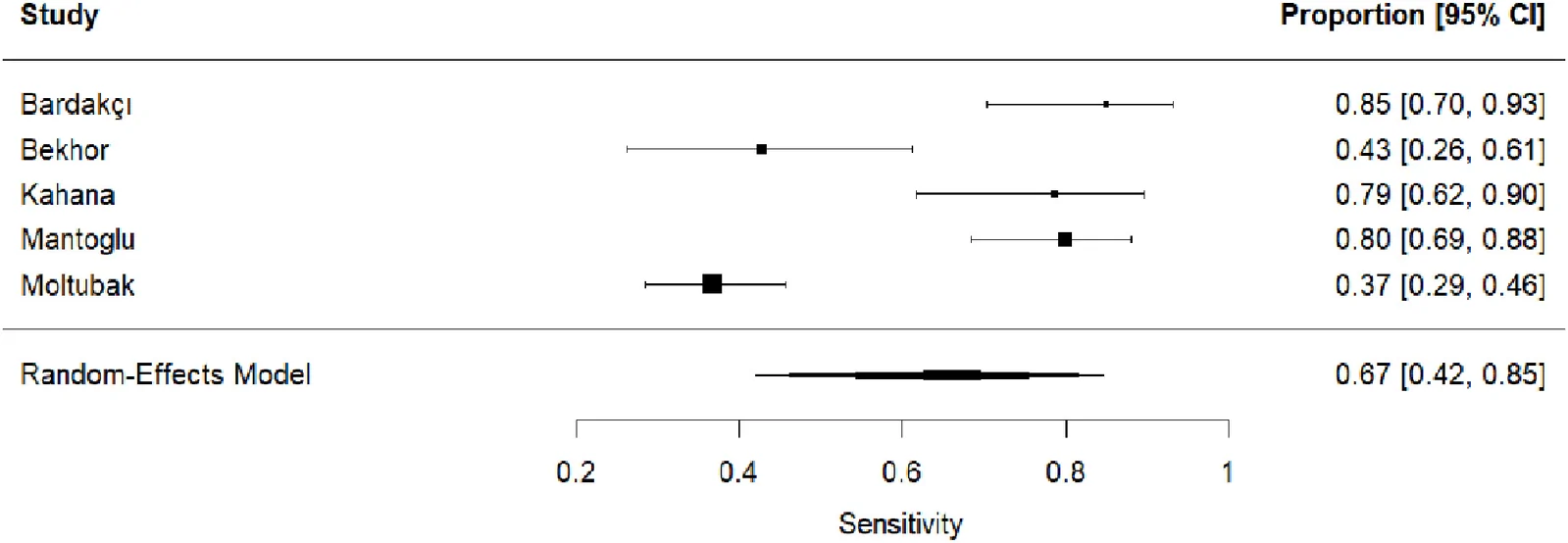
Forest plot of pooled sensitivity of the appendicitis inflammatory response score for diagnosing acute appendicitis during pregnancy.

**Fig. 3:**
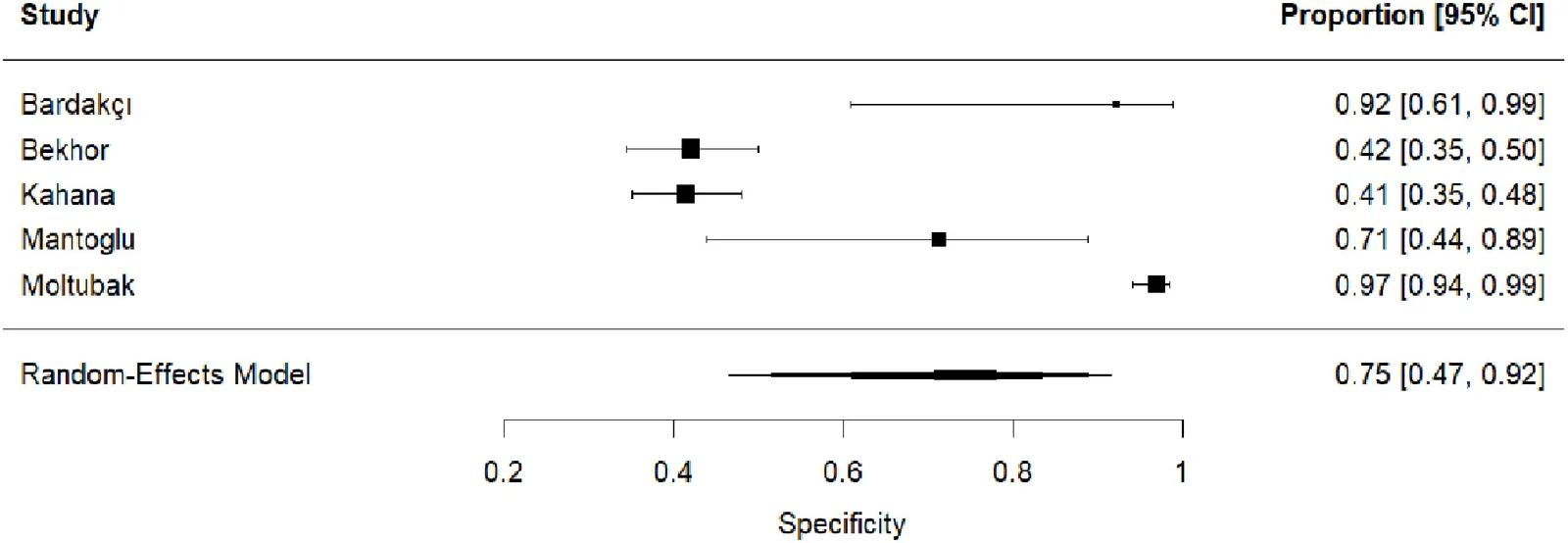
Forest plot of pooled specificity of the appendicitis inflammatory response score for diagnosing acute appendicitis during pregnancy.

### 3.4 Hierarchical summary receiver operating characteristic analysis

A HSROC model based on the bivariate random-effects Reitsma approach yielded an AUC of 0.752, indicating moderate overall diagnostic accuracy. The normalized partial AUC across the observed range of false-positive rates was 0.669. The substantial between-study variability indicates that the summary AUC should be interpreted in the context of the heterogeneous thresholds and study populations (Fig. 4).

**Fig. 4:**
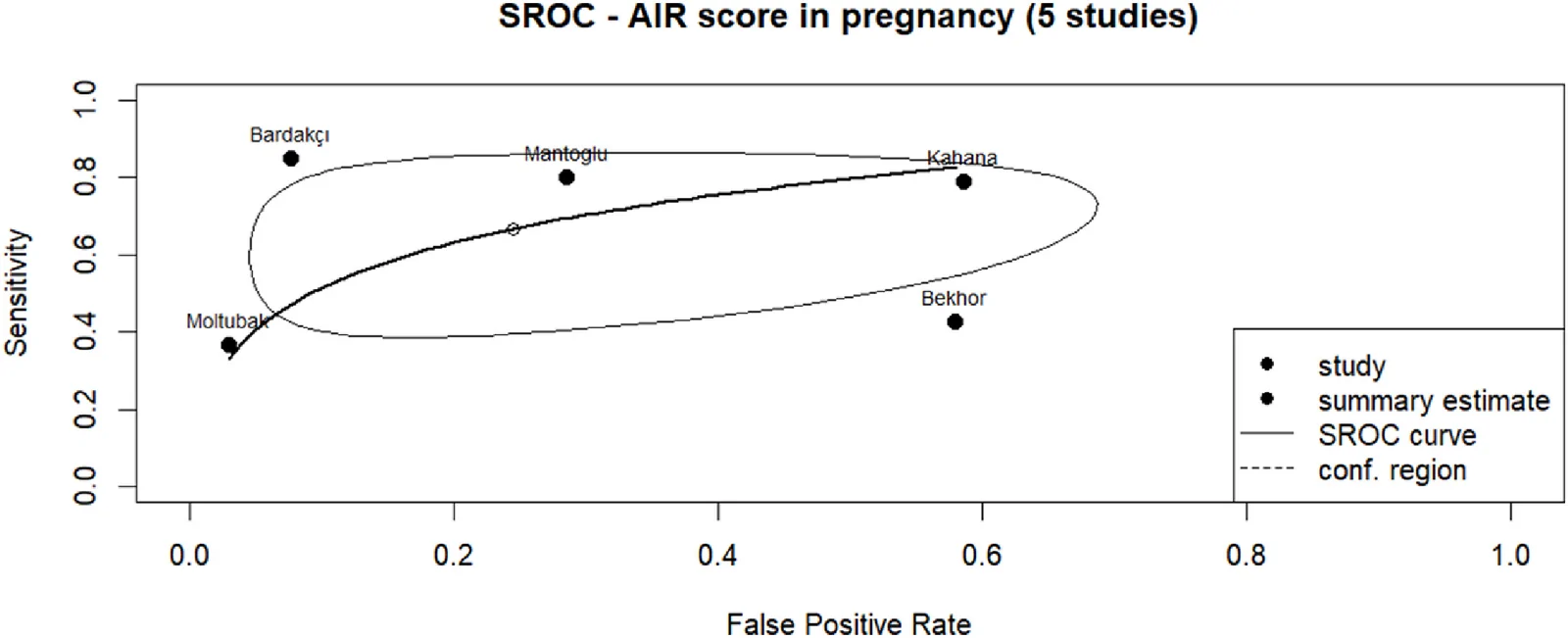
Hierarchical summary receiver operating characteristic (HSROC) curve of the appendicitis inflammatory response score for diagnosing acute appendicitis during pregnancy.

### 3.5 Heterogeneity assessment

The univariate analyses demonstrated substantial heterogeneity, with I² values of 92.0% for sensitivity and 96.4% for specificity. In the bivariate model, the Zhou–Dendukuri estimate of overall heterogeneity was 75.6%, while Holling estimates ranged from 85.8% to 92.6% before sample-size adjustment and from 12.9% to 13.8% after adjustment. These findings support considerable between-study variability and prompted exploratory analyses of threshold, prevalence, and reference-standard effects.

Cut-off-stratified analyses further demonstrated the threshold effect. Studies using lower cut-offs of 5–6 had pooled sensitivity of 69.2% (95% CI: 43.5%–86.8%) and specificity of 44.3% (95% CI: 35.8%–53.1%), whereas studies using a high cut-off of ≥9 had sensitivity of 63.7% (95% CI: 15.8%–94.2%) and specificity of 96.7% (95% CI: 93.8%–98.3%). Specificity heterogeneity was 54.0% in the 5–6 subgroup and 0% in the ≥9 subgroup, although the latter estimate was based on only two studies and therefore requires cautious interpretation. Within Moltubak et al. [8], using the same 386-patient cohort and reference standard, lowering the threshold from ≥9 to ≥4 increased sensitivity from 36.8% to 99.1% but reduced specificity from 97.0% to 42.4%, providing direct within-study evidence of the sensitivity–specificity trade-off associated with the AIR threshold.

In reference-standard subgroup analyses, pathology-based studies yielded a pooled sensitivity of 69.6% (95% CI: 32.6%–91.6%) and specificity of 91.1% (95% CI: 62.1%–98.4%), whereas MRI/proxy-based studies yielded a sensitivity of 62.3% (95% CI: 25.6%–88.8%) and specificity of 41.7% (95% CI: 36.8%–46.8%). Reference-standard category was not significantly associated with sensitivity (P = 0.791) but was significantly associated with specificity (QM = 26.25, P < 0.001). Excluding Kahana [7] did not materially resolve heterogeneity: pooled sensitivity was 63.4% (95% CI: 35.1%–84.7%; I² = 93.0%) and specificity was 83.3% (95% CI: 33.9%–98.0%; I² = 96.9%).

Study-level prevalence was not a statistically significant moderator of either sensitivity (QM = 1.72, P = 0.189) or specificity (QM = 1.05, P = 0.306). In the multivariable model including cut-off and prevalence, neither moderator significantly explained sensitivity heterogeneity (QM = 2.04, P = 0.360), while cut-off remained strongly associated with specificity (P < 0.001) and prevalence did not (P = 0.858).

Meta-regression of AIR cut-off as a continuous moderator showed no significant association with sensitivity (QM = 0.013, P = 0.911), whereas cut-off was strongly associated with specificity (QM = 111.42, P < 0.001; coefficient = 0.933, 95% CI: 0.760–1.106). After adjustment for reference-standard category, the association between cut-off and specificity remained significant (coefficient = 0.821, 95% CI: 0.375–1.266; P = 0.0003), whereas reference-standard category was not significant in this model (P = 0.593).

### 3.6 Quality assessment

We used the QUADAS-2 tool to appraise methodological quality (Table 3). Patient selection was the domain with the greatest risk of bias, judged high risk in four studies (Bardakçı, Mantoglu, Bekhor, Kahana) because each restricted enrollment to a selected subgroup—either patients who had already undergone surgery or patients referred for MRI after an inconclusive ultrasound—and low risk only in Moltubak et al. [8], which sampled an unselected pregnant population. For the index test domain, all five studies were rated unclear, since none explicitly reported whether the AIR score was calculated or interpreted blinded to the final diagnosis. The reference standard domain was low risk in three studies that used histopathology for both cases and non-cases (Bardakçı, Mantoglu, Moltubak), but high risk in the remaining two: in Kahana et al. [7], the comparator for the non-operated group was the MRI finding itself rather than histopathology, and in Bekhor et al. [11], the non-operated comparator was ascertained by absence of further healthcare contact rather than pathology. Flow and timing was low risk in the three pathology-based studies and unclear in Bekhor and Kahana, reflecting the same reference-standard ambiguity. On applicability, every study was judged low concern for patient selection and index test, but Bekhor and Kahana were judged high concern for reference standard, since their comparator does not correspond to the accepted diagnostic standard for appendicitis. Taken together, patient selection and reference standard were the domains of greatest concern, and both align closely with the two sources of heterogeneity identified in the meta-regression above (Table 3).

**Table 3:** Quality assessment of included studies.

Study	RISK OF BIAS				APPLICABILITY CONCERNS		
	Patient Selection	Index Test	Reference Standard	Flow and Timing	Patient Selection	Index Test	Reference Standard
Bardakçı et al. (2023)	High	Unclear	Low	Low	Low	Low	Low
Mantoglu et al. (2020)	High	Unclear	Low	Low	Low	Low	Low
Bekhor et al. (2025)	High	Unclear	High	Unclear	Low	Low	High
Kahana et al. (2024)	High	Unclear	High	Unclear	Low	Low	High
Moltubak et al. (2024)	Low	Unclear	Low	Low	Low	Low	Low

## 4 Discussion

This meta-analysis of five studies found that the AIR score has moderate diagnostic performance for AA during pregnancy, with a pooled sensitivity of 66.7% (95% CI: 44.2%–83.5%), a pooled specificity of approximately 75.5% (95% CI: 40.0%–93.5%), and an AUC of 0.752, indicating moderate overall discriminative ability. The AIR score should be regarded as an adjunctive risk-stratification tool rather than a stand-alone diagnostic test, and its interpretation should be integrated with the patient's symptoms, clinical course, physical examination, laboratory findings, and appropriate imaging.

The diagnostic performance of the AIR score observed in the present study appears lower than that reported in non-pregnant populations. Previous validation studies have demonstrated excellent discriminative ability of the AIR score, with reported AUC values ranging from 0.92 to 0.96, compared with 0.72–0.88 for the Alvarado score [1,13,14]. In a recent prospective comparative study, the AIR score achieved an AUC of 0.94, while the adult appendicitis score (AAS) demonstrated the highest overall performance, with an AUC of 0.99 [13]. Similarly, another comparative study reported an AUC of 0.91 for the AIR score, confirming its consistently high diagnostic accuracy in adults presenting with suspected AA [15]. Reflecting this evidence, the 2020 WSES Jerusalem guidelines recommend the AIR score as one of the preferred clinical prediction tools for the initial risk stratification of patients with suspected AA [5].

The reduced diagnostic performance of the AIR score during pregnancy is likely attributable to the physiological and anatomical changes associated with gestation, which directly influence several of its component variables. Physiological leukocytosis is a well-recognized feature of normal pregnancy, with white blood cell counts progressively increasing across gestation and frequently exceeding the AIR score thresholds even in the absence of appendicitis, thereby reducing the specificity of this parameter [1,2]. Likewise, CRP, another key component of the AIR score, may be elevated as a result of normal pregnancy-related inflammatory responses or obstetric and non-obstetric conditions such as preeclampsia, urinary tract infection, and preterm labor, limiting its diagnostic discrimination [3,4]. In addition, progressive enlargement of the uterus displaces the appendix superiorly, resulting in atypical pain localization and less reliable right iliac fossa tenderness or rebound tenderness, which constitute major clinical components of the AIR score [5,14]. Collectively, these physiological and anatomical alterations diminish the discriminatory value of both clinical signs and inflammatory biomarkers, thereby reducing the overall diagnostic accuracy of the AIR score in pregnant women [5,6].

To our knowledge, this is the first diagnostic meta-analysis of the AIR score specifically in pregnancy, and a particular strength is that its sources of heterogeneity were investigated formally, through continuous meta-regression on cut-off, prevalence, and reference-standard category, rather than inferred solely from descriptive subgroup comparison. Several limitations nonetheless warrant caution. Only five studies met eligibility criteria, limiting statistical power and making every meta-regression exploratory; the ≥9 cut-off subgroup in particular rests on just two studies, so its high specificity should not be taken as definitive proof of an optimal pregnancy-specific threshold. All included studies were retrospective and observational, carrying inherent selection and verification biases, and the small number of studies precluded any reliable formal assessment of publication bias. Prevalence's lack of significance may reflect genuinely limited spectrum effect or simply insufficient power to detect it with five studies.

## 5 Conclusion

In conclusion, the AIR score demonstrates moderate overall diagnostic performance for acute appendicitis during pregnancy, with a pooled sensitivity of 66.7%, specificity of approximately 75.5%, and AUC of 0.752. However, substantial heterogeneity limits the interpretation and generalizability of these pooled estimates. The revised analyses indicate that AIR cutoff is an important determinant of specificity, while differences in patient selection and reference-standard pathways likely contribute additional clinical and methodological variability. The AIR score should therefore be used as an adjunctive risk-stratification instrument within a comprehensive clinical and imaging-based diagnostic pathway rather than as a stand-alone diagnostic test. Larger prospective multicenter studies are needed to establish standardized, pregnancy-specific thresholds and to determine how best to integrate the AIR score into diagnostic decision-making.

## Ethical approval

Not applicable.

## Consent to participate

Not applicable.

## Funding

None.

## Data availability

No datasets were generated or analysed during the current study.

## CRediT authorship contribution statement

**Yasaman Tavakoli:** Conceptualization. **Behdad Ghanami:** Data curation. **Azad Alimohammadi:** Software. **Arefeh Motaharnia:** Validation.

## Declaration of competing interest

The authors declare that they have no known competing financial interests or personal relationships that could have appeared to influence the work reported in this paper.
