# Supplementary material for: Diagnostic accuracy of the appendicitis inflammatory response (AIR) score for acute appendicitis during pregnancy: A systematic review and diagnostic meta-analysis
Source: Surg Pract Sci. 2026 Oct 7;27:100367. doi: 10.1016/j.sipas.2026.100367 (PMC13675802; doi:10.1016/j.sipas.2026.100367)
Supplement: Supplementary file 1 [file mmc1.docx]

| **Database** | **Search strategy** | **Results** | **Date** |
| --- | --- | --- | --- |
| **Pubmed** | ((((((((((((((((((((AIR score[tiab]) OR (AIRscore[tiab])) OR (appendicitis inflammatory response[tiab])) OR (inflammatory response score[tiab])) OR (scor*[tiab])) OR (Risk Assessment[Mesh])) OR (risk adjustment[tiab])) OR (risk analys*[tiab])) OR (risk appraisal[tiab])) OR (risk evaluation[tiab])) OR (risk assessment*[tiab])) OR (clinical decision rule[Mesh])) OR ((clinical[tiab]) AND (model*[tiab]))) OR ((clinical[tiab]) AND (score*[tiab]))) OR ((decision[tiab]) AND (rule*[tiab]))) OR ((prediction[tiab]) AND (rule*[tiab]))) OR (Severity of Illness Index[Mesh])) OR (severity of disease index[tiab])) OR (severity of illness index[tiab])) OR (Illness Index Severit*[tiab])) AND (((((Appendicitis[Mesh]) OR (appendicitis[tiab])) OR (appendiceal inflammation[tiab])) OR (appendicular inflammation[tiab])) OR (appendix inflammation[tiab])) AND (((((((((Pregnancy[Mesh]) OR (child*bearing[tiab])) OR (gravidity[tiab])) OR (labor presentation[tiab])) OR (labour presentation[tiab])) OR (pregnanc*[tiab])) OR (gestation[tiab])) OR (pregnant people[Mesh])) OR (pregnant*[tiab])) | **119 results** |  |
| **Embase** | ('AIR score':ti,ab OR 'AIRscore':ti,ab OR 'appendicitis inflammatory response':ti,ab OR 'inflammatory response score':ti,ab OR scor*:ti,ab OR 'severity of illness index'/exp OR 'severity of illness index':ti,ab OR 'severity of disease index':ti,ab OR 'illness index severit*':ti,ab OR 'risk assessment'/exp OR 'risk adjustment':ti,ab OR 'risk analysis':ti,ab OR 'risk appraisal':ti,ab OR 'risk evaluation':ti,ab OR 'risk assessment':ti,ab OR 'clinical decision rule'/exp OR 'clinical decision* rule*':ti,ab OR 'clinical prediction* rule*':ti,ab OR (clinical:ti,ab AND model*:ti,ab) OR (clinical:ti,ab AND score*:ti,ab) OR (decision:ti,ab AND rule*:ti,ab) OR (prediction:ti,ab AND rule*:ti,ab)) AND ('appendicitis'/exp OR 'acute appendicitis'/exp OR 'appendicitis':ti,ab OR 'appendiceal inflammation':ti,ab OR 'appendicular inflammation':ti,ab OR 'appendix inflammation':ti,ab) AND ('pregnancy'/exp OR 'pregnant person'/exp OR 'pregnant woman'/exp OR 'child* bearing':ti,ab OR 'gravidity':ti,ab OR 'labor presentation':ti,ab OR 'labour presentation':ti,ab OR 'pregnanc*':ti,ab OR 'gestation':ti,ab OR 'pregnant*':ti,ab) | **256**  **results for search** |  |
| **Scopus** | (TITLE-ABS-KEY ("AIR score" OR "AIRscore" OR "appendicitis inflammatory response" OR "inflammatory response score" OR scor* OR "severity of illness index" OR "severity of disease index" OR "illness index severit*" OR "risk adjustment" OR "risk analysis" OR "risk appraisal" OR "risk evaluation" OR "risk assessment" OR "clinical decision* rule*" OR "clinical prediction* rule*" OR (clinical AND model*) OR (clinical AND score*) OR (decision AND rule*) OR (prediction AND rule*))) AND (TITLE-ABS-KEY ("appendicitis" OR "acute appendicitis" OR "appendiceal inflammation" OR "appendicular inflammation" OR "appendix inflammation")) AND (TITLE-ABS-KEY ("pregnancy" OR "pregnant person" OR "pregnant woman" OR "child* bearing" OR "gravidity" OR "labor presentation" OR "labour presentation" OR "pregnanc*" OR "gestation" OR "pregnant*")) | **276**  **documents found** |  |
| **WOS** | TS=("AIR score" OR "AIRscore" OR "appendicitis inflammatory response" OR "inflammatory response score" OR scor* OR "severity of illness index" OR "severity of disease index" OR "illness index severit*" OR "risk adjustment" OR "risk analysis" OR "risk appraisal" OR "risk evaluation" OR "risk assessment" OR "clinical decision* rule*" OR "clinical prediction* rule*" OR (clinical AND model*) OR (clinical AND score*) OR (decision AND rule*) OR (prediction AND rule*)) AND TS=(appendicitis OR "acute appendicitis" OR "appendiceal inflammation" OR "appendicular inflammation" OR "appendix inflammation") AND TS=(pregnancy OR "pregnant person" OR "pregnant woman" OR "pregnant people" OR "child* bearing" OR gravidity OR "labor presentation" OR "labour presentation" OR pregnanc* OR gestation OR pregnant*) | **112 Documents** |  |
| **TOTAL** |  | **763** | 7 January2026 |
